# Guidance for engagement in health guideline development: A scoping review

**DOI:** 10.1002/cl2.70006

**Published:** 2024-11-25

**Authors:** Jennifer Petkovic, Alison Riddle, Lyubov Lytvyn, Joanne Khabsa, Elie A. Akl, Vivian Welch, Olivia Magwood, Pearl Atwere, Ian D. Graham, Sean Grant, Denny John, Srinivasa Vittal Katikireddi, Etienne V. Langlois, Reem A. Mustafa, Alex Todhunter‐Brown, Holger Schünemann, Airton T. Stein, Thomas W. Concannon, Peter Tugwell

**Affiliations:** ^1^ Bruyère Research Institute University of Ottawa Ottawa Ontario Canada; ^2^ School of Epidemiology and Public Health, Faculty of Medicine University of Ottawa Marmora Ontario Canada; ^3^ Department of Clinical Epidemiology and Biostatistics McMaster University Hamilton Ontario Canada; ^4^ American University of Beirut Medical Center, Clinical Research Institute Beirut Lebanon; ^5^ Department of Internal Medicine American University of Beirut Medical Center Beirut Lebanon; ^6^ Methods Centre, Bruyère Research Institute Ottawa Ontario Canada; ^7^ C.T. Lamont Primary Health Care Research Centre, Bruyere Research Institute Ottawa Ontario Canada; ^8^ Bruyere Research Institute Ottawa Ontario Canada; ^9^ School of Epidemiology, Public Health and Preventative Medicine University of Ottawa Ottawa Ontario Canada; ^10^ HEDCO Institute for Evidence‐Based Educational Practice University of Oregon Eugene Oregon USA; ^11^ Campbell Collaboration New Delhi India; ^12^ MRC/CSO Social and Public Health Sciences Unit University of Glasgow Glasgow UK; ^13^ Alliance for Health Policy and Systems Research, World Health Organization Geneva Switzerland; ^14^ Department of Internal Medicine and Population Health University of Kansas Medical Centre Kansas City Kansas USA; ^15^ Nursing, Midwifery and Allied Health Professions, Research Unit Glasgow Caledonian University Glasgow UK; ^16^ Departments of Health Research Methods, Evidence, and Impact and of Medicine McMaster University Hamilton Ontario Canada; ^17^ Department of Public Health Universidade Federal de Ciências da Saúde Porto Alegre Brazil; ^18^ The RAND Corporation Boston Massachusetts USA; ^19^ Department of Medicine University of Ottawa Ottawa Ontario Canada; ^20^ Clinical Epidemiology Program Ottawa Hospital Research Institute Ottawa Ontario Canada

**Keywords:** engagement, evidence synthesis, guidelines, stakeholders

## Abstract

**Background:**

Health guideline developers engage with interested people and groups to ensure that guidelines and their recommendations are relevant and useful to those who will be affected by them. These ‘interest‐holders’ include patients, payers/purchasers of health services, payers of health research, peer review editors, product makers, programme managers, policymakers, providers, principal investigators, and the public. The Guidelines International Network (GIN) and McMaster University Guideline Development Checklist describes 146 steps of the guideline process organized into 18 topics. While one topic focuses on engagement, it does not describe how to engage with interest‐holders. In addition, interest‐holder input could be sought throughout the guideline development process. This scoping review is part of a series of four related reviews. The three other reviews address barriers and facilitators to engagement in guideline development, managing conflicts of interest in guideline development, and assessing the impact of interest‐holder engagement on guideline development. The four reviews will inform the development of guidance for multi‐interest‐holder engagement in guideline development; the GIN‐McMaster Guideline Development Checklist Extension for Engagement.

**Objectives:**

The objective of this scoping review is to identify, describe, and summarise existing guidance and methods for multi‐interest‐holder engagement throughout the health guideline development process.

**Search Methods:**

We conducted one comprehensive search for studies of engagement in guidelines to meet the inclusion criteria of one or more of the four systematic reviews in this series. We searched MEDLINE (OVID), CINAHL (EBSCO), EMBASE (OVID), PsycInfo (OVID) and SCOPUS databases up to September 2022. We did not include limits for date, study design, or language. We searched websites of agencies and organizations that engage interest‐holder groups, such as the Agency for Healthcare Research and Quality (AHRQ), CIHR Strategy for Patient‐Oriented Research (SPOR), National Institute for Health and Care Research (NIHR) Be Part of Research, Guidelines International Network (G‐I‐N), the National Institute for Health and Care Excellence, and the PatientCentred Outcomes Research Institute (PCORI). We handsearched the websites of guideline producing agencies. We solicited additional grey literature from the members of the MuSE Consortium.

**Selection Criteria:**

Studies were included in this review if they reported on engagement of any of our identified groups, patients, payers/funders of research, payers/purchasers of health services, policymakers, programme managers, providers, principal investigators/researchers, peer review editors, product makers in the development of a health guideline. Titles and abstracts of identified citations were screened independently, in duplicate. The full text of potentially relevant papers were screened for eligibility into one or more of the four reviews in the series. Screening was done independently, by two reviewers. The team held weekly meetings with all reviewers involved in screening to discuss and resolve conflicts.

**Data Collection and Analysis:**

Two reviewers extracted relevant data into a pilot‐tested data extraction form using Excel. We used the GIN‐McMaster guideline development checklist as a framework for extracting the available guidance for each of our identified interest‐holder groups throughout the development process. We presented descriptive statistics of the number of papers reporting guidance for each groups across the steps of the guideline process. We synthesized the relevant text using a qualitative meta‐summary approach.

**Main Results:**

We included 16 papers (from 17 reports). These papers were from Australia, Denmark, the Netherlands, the UK, and the USA, and eight papers were international (countries not specified). The papers provided guidance for at least one of our interest‐holder groups for at least one stage of guideline development. We mapped this guidance to the GIN‐McMaster Guideline Development Checklist to identify the available guidance for each of our interest‐holder groups across all stages of the guideline development process. Guidance was available for patient engagement in 15 of the 16 papers. At least two papers provided guidance for each of the 18 topics of the GIN‐McMaster Guideline Development Checklist. For healthcare providers, 9 papers provided guidance for their engagement across 10 of the 18 guideline development topics. Guidance for engaging with the public was provided for 14 of the 18 topics and reported in 4 of our included papers. For payers/purchasers of health services, policymakers, product makers, programme managers, and principal investigators, 2–3 papers provided guidance for these groups across 4–7 topics of the GIN‐McMaster checklist. We did not identify any specific guidance for payers of health research or for editors of peer‐reviewed journals.

**Authors' Conclusions:**

Guidance for interst‐holder engagement in guidelines is available but has focused primarily on patients. We will utilize the guidance identified in this scoping review to inform the GIN‐McMaster Guideline Development Checklist Extension for engagement. Combined with the information obtained from the other systematic reviews in this series, we will address the gaps in guidance for the other identified interest‐holder groups.

## 1 PLAIN LANGUAGE SUMMARY GUIDANCE FOR INTEREST‐HOLDER ENGAGEMENT IN HEALTH GUIDELINE DEVELOPMENT

### 1.1 What is this review about?

There are gaps in literature about when and how to engage with different interest‐holders throughout the guideline development process. Interest‐holder engagement helps to make sure that the guideline will meet the needs of the people and groups who are affected by the guideline. Interest‐holders include patients, the public, providers of health services, health programme managers, and many more. This review identifies, describes and summarizes guidance and methods for engagement. We mapped this guidance to the GIN‐McMaster Guideline Development Checklist to summarize the available guidance for each of our interest‐holder groups.

### 1.2 What are the main findings of this review?

#### 1.2.1 What studies are included

This review includes 16 papers that provide guidance for at least one interest‐holder group and at least one topic of guideline development. It includes papers published from 1996 to 2022 and in Australia, Denmark, the Netherlands, the UK, and the USA, and eight international papers.

#### 1.2.2 How much guidance is available for the different interest‐holder groups under review?

A majority of the guidance focuses on patient involvement, with limited guidance for healthcare providers, policymakers, product makers, programmes managers, and payers of health services. We did not identify guidance for funders of health research or editors of peer‐reviewed journals.

#### 1.2.3 What is the available guidance for multi‐interest‐holder engagement throughout the steps of health guideline development?

The earlier topics in the guideline development process, such as organization, planning, training, and budget, priority setting, guideline group membership and processes, and topic selection have the most guidance. Conflict of interest considerations, question generation, and considering importance of interventions and outcomes, judging quality or certainty of a body of evidence, developing recommendations, and updating have less guidance. There is no specific guidance on the optimal number of guideline panel members, probably because this is dependent on the context and setting of the guideline, and available time and resources.

### 1.3 What do the findings of this review mean?

This review identifies gaps in guidance for engaging with interest‐holders throughout the guideline and recommendation development process. We found limited guidance for all interest‐holder groups except for patients and most guidance referred to the early stages of guideline development.

We will use the findings of this review to develop an extension of the GIN‐McMaster Guideline Development Checklist focused on interest‐holder engagement.

## 2 BACKGROUND

### 2.1 The problem, condition or issue

Patient care, public health, and health systems decisions are informed by guidelines which evaluate and summarize the available evidence. They weigh the benefits and risks while assessing the acceptability, feasibility, and potential equity considerations of available care and policy options (Institute of Medicine US, 2011). The involvement of individuals and groups who are affected by the recommendations included in these guidelines is important for ensuring that the right questions are asked and different, potentially competing considerations are weighed appropriately (Gillard et al., 2012; Oliver et al., 2014). Interest‐holder engagement can improve the relevancy, transparency, and usefulness of guidelines and improve adherence to the treatments or practices recommended (Carroll, 2017; Esmail et al., 2015; Schunemann et al., 2014).

For guidelines, there are many potentially interested people and groups; for the purposes of this work, we refer to these groups as ‘interest‐holders’. We previously used the term ‘stakeholder’ but given its historical meaning related to colonialism, we no longer use this term (Akl et al., 2024). Types of interest‐holders include patients, payers/purchasers of health services, payers of health research, peer review editors, product makers, programme managers, policy makers, providers, principal investigators, and the public (Concannon et al., 2012; Tugwell et al., 2006). To date, clinicians and other healthcare providers as well as patients or consumers have been the most often engaged in guideline development; while other interest‐holders have been less frequently included (Armstrong & Bloom, 2017; Lavis et al., 2008; Oxman et al., 2006; van de Bovenkamp & Zuiderent‐Jerak, 2015). Other terms may be used to refer to ‘engagement’ such as involvement, collaboration, or partnership (Hoddinott et al., 2018). For this project, we use the term ‘interest‐holder engagement’.

There are many frameworks for guideline development; engagement is often included as one step of the process. However, details on how to engage with interest‐holders may be lacking. As engagement has become widely accepted as a critical part of guideline development (Institute of Medicine US, 2011; Qaseem, 2012; Schunemann et al., 2006), this review aims to identify and describe guidance for interest‐holder engagement. We use the GIN‐McMaster Guideline Development Checklist to identify the stages of guideline development (Schunemann et al., 2014). The GIN‐McMaster Checklist describes 146 steps of guideline development organized into 18 non‐sequential topics. While interest‐holder involvement is included as a topic in the checklist, we have identified guidance for engagement throughout all 18 topics (Schunemann et al., 2014).

### 2.2 Definitions

For this work, we use the following definitions:

- guidelines are ‘systematically developed evidence‐based statements which assist providers, recipients and other interest‐holders to make informed decisions about appropriate health interventions’ (World Health Organization, 2003) interest‐holders groups;
- include 'interest‐holders', those involved in and/or affected by the guideline in question; for this project, these are classified as follows: patients, the public, providers, payers/purchasers of health services, payers of research, product makers, policymakers, programme managers, peer review editors, principal investigators (Akl et al., 2024; Concannon et al., 2012; Tugwell et al., 2006);
- engagement is an approach to ensure the contribution of interest‐holders towards the development of the guideline, completion of any of the stages of the guideline, or dissemination of the guideline and its recommendations (Frank et al., 2020; Pollock et al., 2018);
- we defined guidance as systematic approaches for interest‐holder engagement (Armstrong, Rueda, et al., 2017) and we include descriptions of process(es), checklists, concepts, models, outlines, systems, plans and/or overviews on engaging interest‐holders in guideline development processes.

### 2.3 Description of the phenomena of interest

Interest‐holder engagement is helpful for guideline development to ensure that diverse opinions or preferences have been considered to increase the knowledge available for decision‐making (Oliver et al., 2018). Some interest‐holder groups may be more intensely involved at certain steps of the process than others (Crowe, 2017; Oliver et al., 2008; Pollock et al., 2019). The stage at which interest‐holder groups are included may affect the guideline development process. For example, including peer review editors early on in the development can ensure that the guideline produced follows the standards required by the journals that may publish it. Ensuring that patients are included when defining the scope or the research question ensures that the right concerns are being addressed. For example, patients can introduce novel topics to the guideline development process, provide important context, or suggest additional outcomes of importance (Armstrong et al., 2018, 2020; Díaz del Campo et al., 2011).

**Figure 1:**
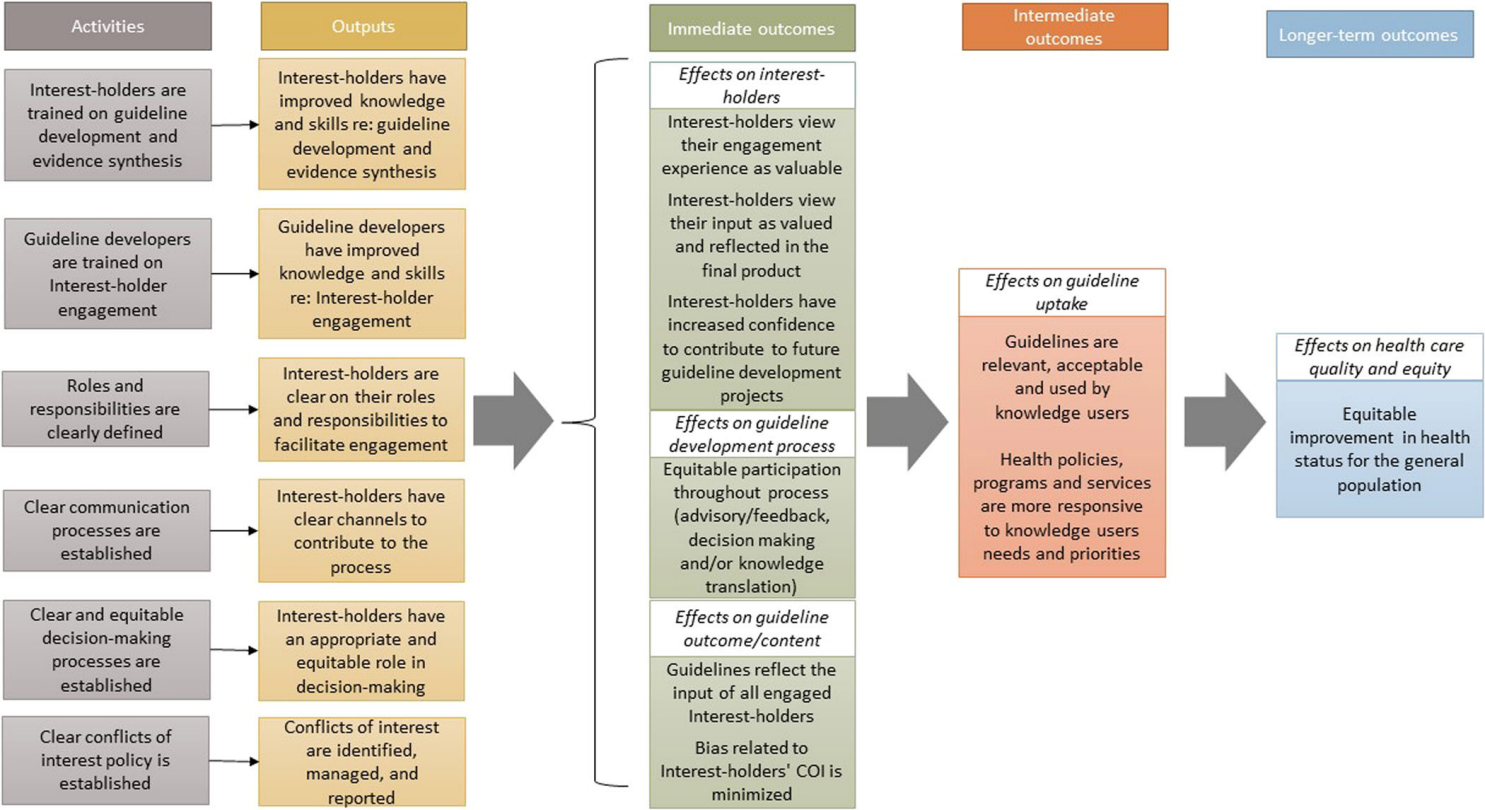
Logic model of the effects of engagement in guideline development.

Interest‐holders may have different roles for different aspects of the guideline development. For example, one group may provide feedback or advice for a stage of the process but be engaged in a decision‐making capacity for another. Engagement is a complex process and requires certain activities to be effective, which we portray in a logic model produced for the overall MuSE project (Figure 1). This scoping review focuses on identifying and summarizing the available guidance for engaging with interest‐holders according to the 18 topics of the GIN‐McMaster Guideline Development Checklist (Schunemann et al., 2014).

### 2.4 Why it is important to do the review

While the importance of engagement in guidelines has been well recognized, there is a need to identify guidance for how and when to engage multiple interest‐holders throughout each step of the process (Qaseem, 2012). Guidance for single interest‐holder groups exists, particularly patients, caregivers, consumers, or service users, such as Armstrong and colleagues' 10‐step model for engaging patients in guideline development (Armstrong, Rueda, et al., 2017). Additionally, Schunemann et al. reviewed 35 guideline manuals (from 2003 to 2012) to develop the GIN‐McMaster Guideline Development Checklist (Schunemann et al., 2014). The underlying work for that checklist is currently being updated to assess the guidance available in handbooks of guideline developing organizations.

The GRADE (Grading of Recommendations Assessment, Development and Evaluation) system is internationally recognised as a standard for guideline development (Guyatt et al., 2008). The GRADE Handbook recommends that ‘the guideline panel and supporting groups … work collaboratively, informed through consumer and [interest‐holder] involvement’ (Schunemann et al., 2013). It does not provide guidance on how this should be achieved. The MuSE (formerly the Multi‐Stakeholder Engagement) Consortium is an international network of interest‐holders interested in engagement in research (Concannon et al., 2019; Petkovic et al., 2020). Established in 2015, the group has over 140 members from 21 countries and represents all of our identified interest‐holder groups. One goal of this group is to develop guidance for engagement in guideline development. To develop this guidance, the group conducted this scoping review to identify and summarize existing guidance for interest‐holder engagement in guideline development while also conducting three other reviews focused on (a) barriers and facilitators to engagement (Magwood et al., 2022), (b) disclosure, management and reporting of potential conflicts of interest during guideline development (Khabsa et al., 2022) and (c) the impact of engagement on the guideline development process. The results of these four reviews will inform the development of an extension to the GIN‐McMaster Guideline Development Checklist that focuses on how interest‐holders can be engaged in each stage of the process. The Engagement Extension checklist may be used to assist organisations who develop healthcare, public health, and health policy guidelines, such as the World Health Organization, to involve multiple interest‐holders in the guideline development process to ensure the development of relevant, high quality, and transparent guidelines.

## 3 OBJECTIVES

This review aims to identify, describe, and summarise existing guidance and methods for interest‐holder engagement throughout the health guideline development process.

## 4 METHODS

We had planned to use thematic synthesis to combine the guidance for each step. However, in discussion with the authors, we decided to use a qualitative meta‐synthesis approach as this was better suited for presenting the information in the matrix, as planned.

We had also planned to conduct quality appraisal of our included papers using the practicality, relevancy, and legitimacy criteria as described by Movsisyan et al. (2019). Guidance for scoping reviews suggests that the utility of critical or quality appraisal for scoping reviews is uncertain and that they should only be conducted when there is a strong rationale for it (Levac et al., 2010; Peters et al., 2020; Pollock et al., 2021). We aimed to present the available guidance for each interest‐holder group throughout the 18 topics of guideline development. Therefore, rating our papers using the Movsisyan criteria did not add anything to our presentation of the literature as we did not intend to assess each guidance document but specifically the individual guidance items as they related to the different interest‐holder groups.

### 4.1 Criteria for including and excluding studies

#### 4.1.1 Types of study designs

We included methodology studies describing the development of guidance for engagement in guideline development. To be included, papers had to describe a process and methods for interest‐holder engagement in guideline development, using the definition of guideline described above. Quantitative, qualitative, and mixed‐method studies were eligible. We included case reports if they provided guidance based on experience with engagement in a guideline. We also included narrative reviews summarizing guidance, for example, for particular guideline developing organizations. We excluded editorials, commentaries, protocols, and conference abstracts.

Our methods followed the guidance for scoping reviews described by Arksey and O'Malley (2005) and the PRISMA Extension for Scoping Reviews (Tricco et al., 2018). The Arkey and O'Malley framework for scoping reviews includes: (1) identifying the research question, (2) identifying relevant studies, (3) study selection, (4) charting the data, and (5) collating, summarizing, and reporting results (Arksey and O'Malley, 2005).

#### 4.1.2 Types of participants

We have previously identified 10 groups of interest‐holders whose input can enhance the relevance and uptake of research (Concannon et al., 2012; Concannon et al., 2019; Tugwell et al., 2006). These 10 ‘Ps’ represent all those who would be responsible for or affected by health and healthcare decisions (Concannon et al., 2012) and the 10 Ps were developed based on our previous research (Concannon et al., 2012; Petkovic et al., 2020; Tugwell et al., 2006). For simplicity, we refer to these groups as the 10 ‘Ps’:

- Patients, caregivers, and patient advocates,
- Payers/purchasers of health services,
- Payers of research,
- Peer review editors,
- Policymakers,
- Principal investigators and their research teams,
- Product makers,
- Programme managers,
- Providers of health care, and the Public.

#### 4.1.3 Phenomena of interest

We included papers that described guidance for interest‐holder engagement in the clinical practice or public health guideline development process.

We included papers discussing any topic of the guideline development process as described by the GIN‐McMaster Guideline Development Checklist (Schunemann et al., 2014):

1. Organization, budget, planning and training.
2. Priority‐setting.
3. Guideline group membership.
4. Establishing guideline group processes.
5. Identifying target audience and topic selection.
6. Consumer and Interest‐holder involvement.
7. Conflict of interest considerations.
8. Question generation.
9. Considering importance of outcomes and interventions, values, preferences, and utilities.
10. Deciding what evidence to include and searching for evidence.
11. Summarizing evidence and considering additional information.
12. Judging quality, strength or certainty of body of evidence.
13. Developing recommendations and determining their strength.
14. Wording of recommendations and of considerations about implementation, feasibility and equity.
15. Reporting and peer review.
16. Dissemination and implementation.
17. Evaluation and use.
18. Updating.

#### 4.1.4 Types of settings

We included papers discussing any phase of the guideline development process, including those presenting guidance across all steps as well as those focused on a single step of guideline development.

### 4.2 Search strategy

We developed one comprehensive search strategy for all four systematic reviews in this series in consultation with a medical librarian. Our search strategies were peer‐reviewed by a second medical librarian. We searched: MEDLINE (OVID), CINAHL (EBSCO), EMBASE (OVID), PsycInfo (OVID) and SCOPUS. We did not include limits for date, study design or language. The databases were searched up to September 2022.

We conducted an extensive grey literature search using the websites of agencies who actively engage interest‐holder groups in their work. We searched:

- Agency for Healthcare Research and Quality's (AHRQ) https://www.ahrq.gov/.
- Canadian Institutes of Health Research (CIHR) Strategy for Patient‐Oriented Research (SPOR).
- National Institute for Health and Care Research (NIHR).
- Be Part of Research.
- Guidelines International Network (G‐I‐N).
- INVOLVE https://www.invo.org.uk/.
- National Institute for Health and Care Excellence (NICE).
- Patient‐Centred Outcomes Research Institute (PCORI).
- Australia's National Health Medical Research Council (NHMRC).
- World Health Organization (WHO), including Latin American and Caribbean Health Sciences Literature (LILACS).

We solicited suggestions for additional grey literature sources from the members of the MuSE Consortium.

Backward and forward citation tracking was performed on included articles to identify additional eligible papers. We reviewed the reference lists of relevant reviews to identify eligible papers for inclusion.

### 4.3 Details of study coding categories

Two reviewers independently screened titles and abstracts in duplicate to identify relevant studies meeting the prespecified and pilot‐tested inclusion criteria and then the full text of those identified as potentially relevant were screened independently by two authors using Covidence software (https://www.covidence.org/).

The data extraction form was pre‐tested and refined to ensure consistent understanding across all members of the extraction team. Data were extracted independently, in duplicate by two reviewers using Excel. Disagreements were resolved by discussion and with a third member of the research team when necessary.

We extracted data on the existing guidance for engagement according to the 18 topics and using all 146 individual steps of the GIN‐McMaster checklist (Table 1), as well as:

- General paper characteristics.
- Interest‐holders groups and definition of interest‐holder.
- Definition of engagement.
- Characteristics of interest‐holders panel.
- Methods for engaging/method of communication.
- Frequency of engagement.
- Level of engagement (advisory/feedback or decision‐making).

**Table 1:** Framework for data extraction.

	Interest‐holder groups									
Guideline development topics	Patients, caregivers, patient organizations	Public	Providers	Principal investigators + team	Policy makers	Programme managers	Payers/purchasers of health services	Payers of health research	Peer review editors	Product makers
1. Organization, Budget, Planning and Training										
2. Priority Setting										
3. Guideline Group Membership										
4. Establishing Guideline Group Processes										
5. Identifying Target Audience and Topic Selection										
6. Consumer and Interest‐holder Involvement										
7. Conflict of Interest (COI) Considerations										
8. (PICO) Question Generation										
9. Considering Importance of Outcomes and Interventions, Values, Preferences, and Utilities										
10. Deciding what Evidence to Include and Searching for Evidence										
11. Summarizing Evidence and Considering Additional Information										
12. Judging Quality or Certainty of a Body of Evidence										
13. Developing Recommendations and Determining their Strength										
14. Wording of Recommendations and of Considerations of Implementation, Feasibility, and Equity										
15. Reporting and Peer Review										
16. Dissemination and Implementation										
17. Evaluation and Use										
18. Updating										

*Note*: We extracted the verbatim guidance as reported in the included papers.

### 4.4 Data synthesis

We have presented descriptive statistics reporting the number of papers presenting guidance for each interest‐holder group across the 18 stages of the GIN‐McMaster Guideline Development Checklist. We synthesized the relevant text from our included studies using a qualitative meta‐summary approach (Gates et al., 2020; Ribeiro et al., 2014; Sandelowski et al., 2007). This is a quantitatively oriented approach to synthesizing qualitative findings which allows for reporting the relative frequency of the guidance statements. Guidance was extracted from each paper as reported by the authors and then grouped and edited, where appropriate. We have presented this information as a matrix indicating the existing guidance for each interest‐holder group and for each step of guideline development.

## 5 RESULTS

### 5.1 Description of studies

#### 5.1.1 Results of the search

We conducted a combined search with the other three reviews in this series (Khabsa et al., 2022; Magwood et al., 2022). This broad search (Supporting Information S1; Supporting Information S2; Supporting Information S3; Supporting Information S4; Supporting Information S5; Supporting Information S6) identified 55,364 records. Additional results (*n* = 302) were identified through grey literature. After deduplication using Covidence software, 31,505 records related to engagement and guidelines were assessed for eligibility. We retrieved 731 full‐text papers for review and included 16 reports (from 17 manuscripts). These papers were methodology papers (*n* = 9), case reports of engagement in a specific guideline or guidelines (*n* = 4), and narrative reviews summarizing engagement approaches (*n* = 3) and were from Australia, Denmark, the Netherlands, the UK, the USA, or multiple countries. Table 2 provides a summary of the characteristics of these studies.

**Table 2:** Study characteristics.

References	Country	Publication year	Study design	Interest‐holder groups of the authors	Interest‐holder groups for guidance
Adams (2022)	Australia, Denmark	2022	Methodology Paper	Principal investigators	Patients, Principal investigators, Providers,
Armstrong (2017)	USA	2017	Methodology Paper	Principal investigators	Patients, Public
Bjorkqvist (2021)	United Kingdom and the Netherlands	2021	Methodology Paper	Principal investigators, Payers	Patients
Chalmers (2017)	International (countries not specified)	2017	Case Report	Patients, Providers	Patients
Duff (1996)	United Kingdom	1996	Case Report	Principal investigators	Patients
Eccles (2012)	International (UK, Canada, USA)	2012	Methodology Paper	Principal investigators	Patients, Payers/purchasers of health services, Providers, Product makers
English (2017)	International (Kenya, UK)	2017	Case Report	Principal investigators, Policy Maker	Patients, Policymakers, Providers, Principal investigators
Fretheim (2006)	International (Norway, Italy)	2006	Narrative Review	Principal investigators	Patients, Policymakers, Providers, Programme managers
GIN (2021)	International (countries not specified)	2021	Methodology Paper	Principal investigators, Patients	Patients, Providers, Public
Grant (2021)	USA	2021	Methodology Paper	Principal investigators	Patients
Kelson (2012)	International (countries not specified)	2012	Narrative review	Principal investigators	Patients, Providers
Khodyakov (2020)	USA	2020	Methodology Paper	Principal investigators, Patient advocate	Patients
Kunz (2012)	International (countries not specified)	2012	Narrative review	Principal investigators	Patients, Programme managers, Principal investigators, Product makers, Providers, Public
MacLennan (2017)	International (UK, Sweden, Spain, Belgium, the Netherlands	2017	Methodology Paper	Principal investigators	Patients, Payers/purchasers of health services, Providers
Rapu (2005)	UK	2005	Case Report	Providers	Providers
Wedzicha (2011)	UK	2011	Methodology Paper	Principal investigators	Patients, Providers

#### 5.1.2 Excluded studies

**Figure 2:**
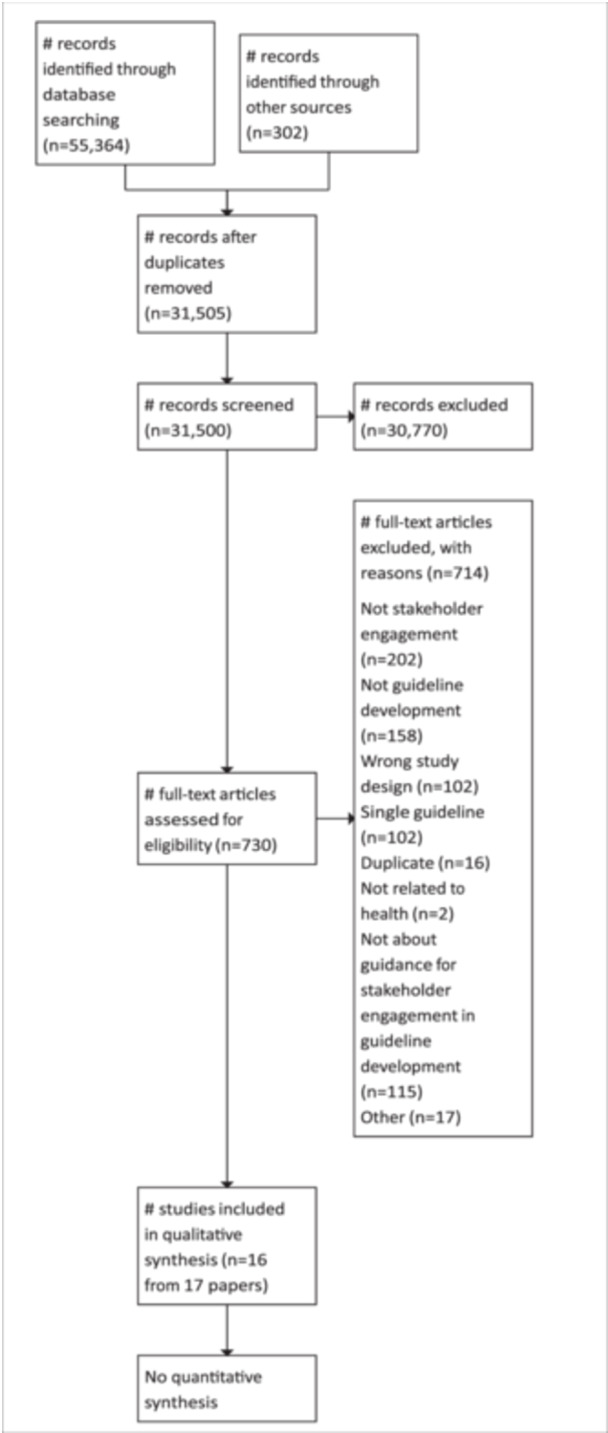
PRISMA‐flow diagram.

We excluded 714 full‐text papers for the following reasons: not related to guideline development, not describing interest‐holder engagement, reporting on a single guideline, not related to health, wrong study design, and not describing guidance for engagement in guideline development (Figure 2).

#### 5.1.3 Synthesis of included studies

**Figure 3:**
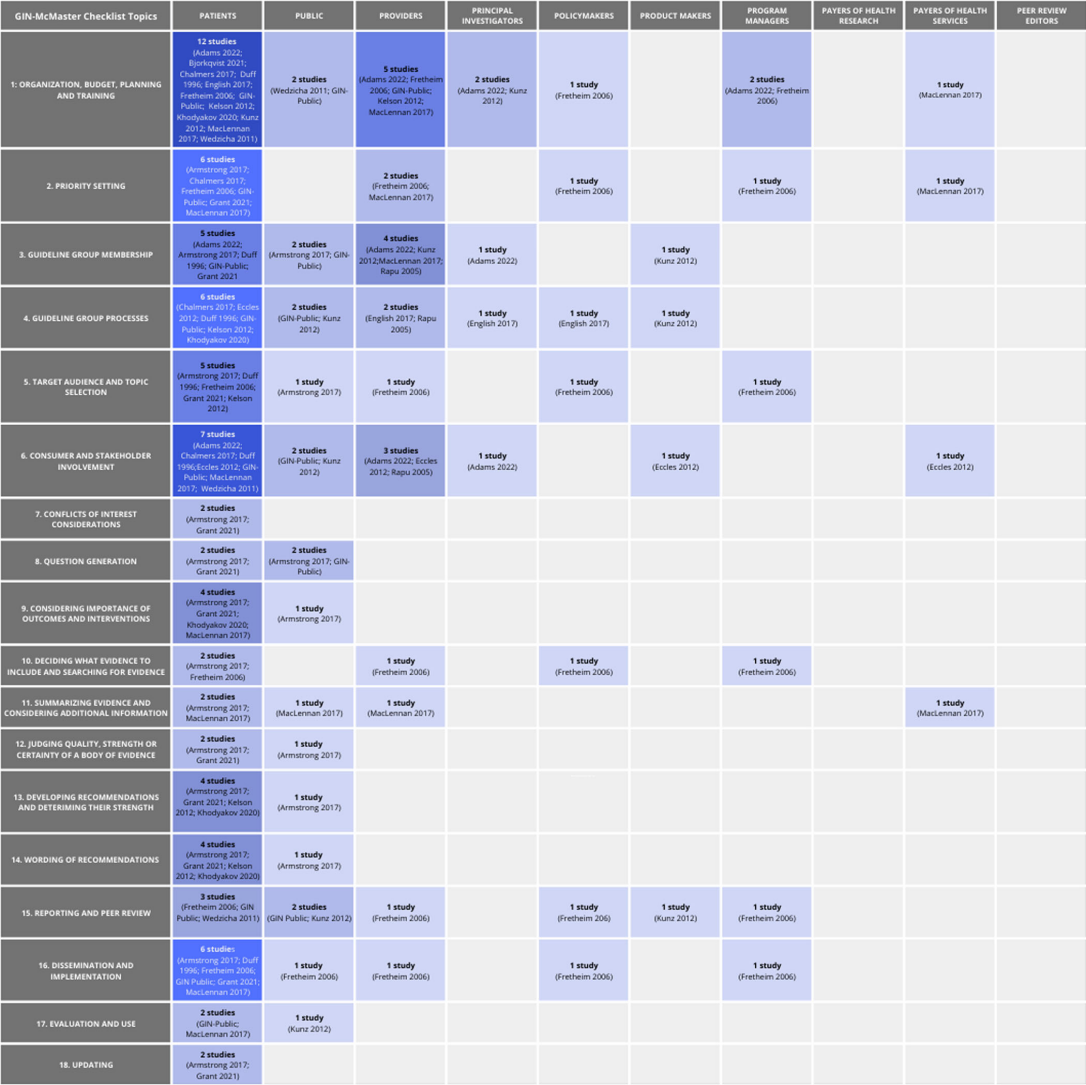
Map of guidance for each interest‐holder group.

We mapped the guidance provided in the 16 included papers to the 18 topics of the GIN‐McMaster checklist (Figure 3). Guidance for patient engagement in guideline development was the most frequently reported. We found existing guidance for patients in at least 2 papers for all 18 topics of the GIN‐McMaster Guideline Development Checklist. Fifteen of the 16 included papers provide guidance for patient engagement in at least 1 stage of guideline development (Adams et al., 2022; Armstrong, Mullins, et al., 2017; Björkqvist et al., 2021; Chalmers et al., 2017; Duff et al., 1996; Eccles et al., 2012; English et al., 2017; Fretheim et al., 2006; GIN, 2021; Grant et al., 2021; Kelson et al., 2012; Khodyakov et al., 2020; Kunz et al., 2012; MacLennan et al., 2017; Wedzicha et al., 2011). At least 2 papers provided guidance for patients for each of the 18 topics of the GIN‐McMaster checklist.

There was guidance for engaging with providers of healthcare for 10 topics of guideline development provided by 9 papers (Adams, 2022; Eccles, 2012; English, 2017; Fretheim, 2006; GIN, 2021; Kelson, 2012; Kunz, 2012; MacLennan, 2017; Rapu, 2005). Guidance for policymakers was reported for seven of the topics provided by two papers (English, 2017; Fretheim, 2006). There was guidance for programme managers for 6 guideline topics provided by two papers (Fretheim, 2006; Kunz, 2012). There was guidance for payers/purchasers of health services for five guideline topics reported by two papers (Eccles, 2012; MacLennan, 2017). Guidance for both principal investigators and product makers was provided for four topics of guideline development. Guidance for engaging with principal investigators or research teams was reported in three papers (Adams, 2022; English, 2017; Kunz, 2012) and two papers provided guidance for product makers (Eccles, 2012; Kunz, 2012). Finally, although there was guidance for engaging with the public for 14 of the 18 topics, this guidance was provided by just 4 of the included papers (Armstrong, 2017; GIN, 2021; Kunz, 2012; Wedzicha, 2011) with 1–2 papers providing guidance for each topic.

There was no specific guidance for payers/funders of health research or editors of peer‐reviewed journals in any of the papers included in this scoping review (see Figure 3).

**Figure 4:**
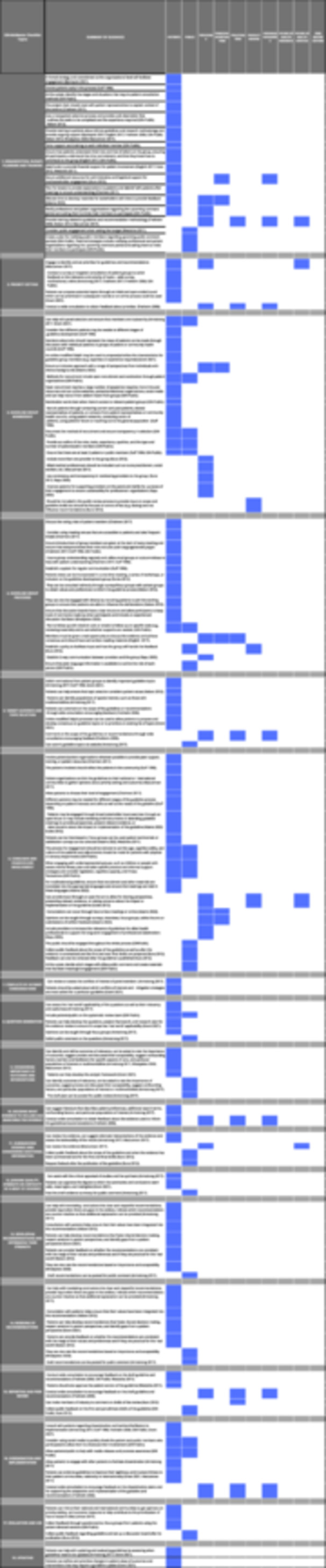
Guidance items by interest‐holder group.

We summarized the guidance provided for each interest‐holder group across the 18 topics of the GIN‐McMaster Guideline Development Checklist in Table 3. A summary of the overlap of guidance for each interest‐holder group is provided in Figure 4.

**Table 3:** Summary of existing guidance.

GIN‐McMaster topic	Guidance
1. Organization, Budget, Planning and Training	**Patients:** A formal strategy and commitment at the organizational level will facilitate engagement (Bjorkqvist, 2021). Involve patients early in the process (Duff, 1996). At the outset, identify the stages and situations that require patient consultation methods (GIN‐Public). The project chair should meet with patient representatives to explain context of discussions (Chalmers, 2017). Use a transparent selection process and provide a job description that outlines the tasks to be completed and the experience required (GIN, 2021; Kelson, 2012). Provide training to patients about clinical guidelines and research methodology and provide ongoing support (Bjorkqvist, 2021; English, 2017; Fretheim, 2006; GIN, 2021; Kelson, 2012; Khodyakov, 2020; MacLennan, 2017). Tailor support and training to each individual member (GIN, 2021). Ensure that patients understand their role and that of others on the group, ensuring all participants understand the time commitment, and that they know how to contribute to the group (English, 2017; GIN, 2021). Ensure that all participants can decide how they will contribute to the clinical guideline (English, 2017; Kelson, 2012). Seek funds to provide financial support for patient involvement (English, 2017; Kunz, 2012; Wedzicha, 2011). Secure additional resources for administrative and logistical support for engagement (Kunz, 2012). Plan for breaks to provide explanations to patients and debrief with patients after meetings to ensure understanding (Chalmers, 2017). Allocate time to develop materials for interest‐holders with time to provide feedback (Adams, 2022). Notify professional and patient organisations regarding the upcoming comment period and asking them to invite their members to participate (Adams, 2022). **Payers of health services:** Provide training related to guideline and recommendation methodology (MacLennan, 2017). **Policymakers:** Provide training related to guideline and recommendation methodology (Fretheim, 2006). **Principal Investigators:** Allocate time to develop materials for interest‐holders with time to provide feedback (Adams, 2022). Secure additional resources for administrative and logistical support for engagement (Kunz, 2012). **Programme Managers:** Provide training related to guideline and recommendation methodology (Fretheim, 2006). Secure additional resources for administrative and logistical support for engagement (Kunz, 2012).
	**Providers:** Provide training related to guideline and recommendation methodology (Fretheim, 2006; Kelson, 2012; MacLennan, 2017). Allocate time to develop materials for interest‐holders with time to provide feedback (Adams, 2022). Notify professional and patient organisations regarding the upcoming comment period and asking them to invite their members to participate (GIN, 2021). **Public:** Consider public engagement when setting the budget (Wedzicha, 2011). Create a plan for notifying public members regarding upcoming public comment periods (GIN, 2021). Potential strategies include notifying professional and patient organisations regarding the upcoming comment period and asking them to invite their members to participate (GIN, 2021).
2. Priority Setting	**Patients:** Engage patients to identify and set priorities for guidelines and recommendations (MacLennan, 2017). Conduct a survey or targeted consultation of patient groups to solicit feedback on the relevance and priority of topics – wide survey, multinational, online (Armstrong, 2017; Chalmers, 2017; Fretheim, 2006; GIN, 2021). Patients can propose potential topics through an initial and open‐ended round which can be prioritized in subsequent rounds or an online process could be used (Grant, 2021). **Payers of health services:** Engage payers to identify and set priorities for guidelines and recommendations (MacLennan, 2017). **Policymakers:** Conduct a wide consultation to obtain feedback about priorities (Fretheim, 2006). **Programme managers:** Conduct a wide consultation to obtain feedback about priorities (Fretheim, 2006). **Providers:** Engage providers to identify and set priorities for guidelines and recommendations (MacLennan, 2017). Conduct a wide consultation to obtain feedback about priorities (Fretheim, 2006).
3. Guideline Group Membership	**Patients:** Can help with panel selection and ensure that members are trustworthy (Armstrong, 2017; Grant, 2021). Decisions about who should represent the views of patients can be made through discussion with individual patients or groups of patient or community health councils (Duff, 1996). Consider that different patients may be needed at different stages of guideline development (Duff, 1996). An online modified Delphi may be used to propose/prioritize the characteristics for guideline group members (e.g., expertise or experience required) (Grant, 2021). Ensure an inclusive approach with a range of perspectives from individuals with diverse backgrounds (Adams, 2022). Methods for recruitment include open recruitment and nomination through patient organizations (GIN, 2021). Open recruitment reaches a large number of people but requires more time and resources and can utilize websites, patient/professional organizations, social media and can help recruit from seldom heard from groups (GIN, 2021). Nomination works best when there is access to relevant patient groups (GIN, 2021). Recruit patients through contacting current and past patients, elected representatives of patients, or contacts from patient representatives or community health councils, using patient networks, contacting carers of patients, using patients' forum or reaching out to the general population (Duff, 1996). Document the method of recruitment and ensure transparency in selection (GIN, 2021). Provide an outline of the roles, tasks, experience, qualities, and the type and number of patient/public members (GIN, 2021). Ensure that there are at least 2 patient or public members (Duff, 1996; GIN, 2021). **Principal Investigators:** Ensure an inclusive approach with a range of perspectives from individuals with diverse backgrounds (Adams, 2022). **Providers:** Include more than one provider in the group (Kunz, 2012). Allied medical professionals should be included such as nurse practitioners, social workers, and so forth (MacLennan, 2017). Use consistency and transparency in nominating providers to the group (Kunz, 2012; Rapu, 2005). Improve systems for supporting providers on the panel and clarify the purpose of their engagement to ensure sustainability for professional organizations (Rapu, 2005). Ensure an inclusive approach with a range of perspectives from individuals with diverse backgrounds (Adams, 2022). **Public:** Can help with panel selection (Armstrong, 2017). Methods for recruitment include open recruitment and nomination through patient organizations (GIN, 2021). Document the method of recruitment and ensure transparency in selection (GIN, 2021). Provide an outline of the roles, tasks, experience, qualities, and the type and number of patient/public members (GIN, 2021). Ensure that there are at least 2 patient or public members (GIN, 2021). **Product Makers:** should be included in the public review process to provide input on scope and guideline drafts but should be focused on errors of fact (e.g., dosing) and not influence recommendations (Kunz, 2012). **All interest‐holders:** Define the remit of the panel and the roles of each place on the panel; specify rules for the process (MacLennan, 2017). Interview potential panel members to ensure they are able to commit to the workload (MacLennan, 2017).
4. Establishing Guideline Group Processes	**Patients:** Discuss the voting roles of patient members (Chalmers 2017). Consider using meeting venues that are accessible to patients and take frequent breaks (Chalmers 2017). Ensure introductions of group members are given at the start of every meeting and ensure that everyone knows their role and uses plain language/avoids jargon (Chalmers 2017; Duff 1996; GIN (2021)). Ensure group understanding regularly and utilize small groups or subcommittees to help with patient understanding (Chalmers, 2017; Duff, 1996). Establish a system for regular communication (Duff, 1996). Patients views can be incorporated in a one‐time meeting, a series of workshops, or inclusion on the guideline development group (Eccles, 2012). They can be consulted indirectly through surveys/focus groups with patient groups to obtain values and preferences to inform the guideline process (Kelson, 2012). They can also be engaged directly by recruiting patients to join the working groups to ensure that patients are able to influence the deliberations (Kelson, 2012). Ensure that discussion boards have a clear structure and allow participants to keep track of comments made by other participants and include an experienced discussion facilitator (Khodyakov, 2020). Plan to follow up with check‐in calls or emails to follow up on specific tasks (e.g., reviewing materials) and to ask whether supports are needed (GIN, 2021). **Policymakers:** Members must be given a real opportunity to discuss the evidence and achieve consensus and should have well‐written reading materials (English, 2017). **Principal Investigators:** Members must be given a real opportunity to discuss the evidence and achieve consensus and should have well‐written reading materials (English, 2017). **Product makers:** Establish a policy to facilitate input and how the group will handle the feedback (Kunz, 2012). **Providers:** Members must be given a real opportunity to discuss the evidence and achieve consensus and should have well‐written reading materials (English, 2017). Establish 2‐way communication between providers and the group (Rapu, 2005). **Public:** Establish a policy to facilitate input and how the group will handle the feedback (Kunz, 2012). Ensure that plain language information is available to outline the role of each person (GIN, 2021). Plan to follow up with check‐in calls or emails to follow up on specific tasks (e.g., reviewing materials) and to ask whether supports are needed (GIN, 2021).
5. Identifying Target Audience and Topic	**Patients:** Solicit nominations from patient groups to identify important guideline topics (Armstrong, 2017; Duff, 1996; Grant, 2021). Patients can help ensure that topic selection considers patient values (Kelson, 2012). Patients can Selection identify populations of special interest, such as those with multimorbidities (Armstrong, 2017). Patients can comment on the scope of the guidelines or recommendations through wide consultation encouraging feedback (Fretheim, 2006). Online modified Delphi processes can be used to allow patients to propose and develop consensus on guideline topics or to prioritize an existing list of topics (Grant, 2021). **Policymakers:** Comment on the scope of the guidelines or recommendations through wide consultation encouraging feedback (Fretheim, 2006). **Programme Managers:** Comment on the scope of the guidelines or recommendations through wide consultation encouraging feedback (Fretheim, 2006). **Providers:** Comment on the scope of the guidelines or recommendations through wide consultation encouraging feedback (Fretheim, 2006). **Public:** can submit guideline topics via website (Armstrong, 2017).
6. Consumer and Interest‐holder Involvement	**Patients:** Involve patient/patient organizations wherever possible to provide peer support, training, or patient resources (Chalmers, 2017). They should be involved throughout the process (GIN, 2021). The patients involved should reflect the patients in the community (Duff, 1996). Patients organizations can link the guidelines to their national or international communities to gather opinions about priority setting and outcomes (MacLennan, 2017). Allow patients to choose their level of engagement (Chalmers, 2017). Different patients may be needed for different stages of the guideline process depending on patient interests and skills as well as the needs of the guideline (Duff, 1996). Patients may be engaged through broad interest‐holder input exercises through an open forum or may include reviewing draft documents or attending guideline meetings to provide perspectives, present relevant evidence, or raise concerns about the impact or implementation of the guideline (Adams, 2022; Eccles, 2012). Patients can be interviewed, or focus groups can be used patient testimonials or satisfaction surveys can be collected (Adams, 2022; Wedzicha, 2011). The process for engagement should be tailored to suit the age, cognitive ability, and culture of the patients and adjustments should be made for patients with physical or sensory impairments (GIN, 2021). When engaging with underrepresented groups, such as children or people with severe mental illness, plan and tailor specific practical and informal support strategies and consider legislation, cognitive capacity, and illness fluctuations (GIN, 2021). For multinational guidelines, ensure that recruitment and other materials are translated into the appropriate languages and ensure that meetings are held in these languages (Adams, 2022). **Payers of health services:** Can provide input through an open forum to allow for sharing perspectives, presenting relevant evidence, or raising concerns about the impact or implementation of the guideline (Eccles, 2012). **Principal Investigators:** Consultations can occur through face‐to‐face meetings or online (Adams, 2022). Opinions can be sought through surveys, interviews, focus groups, online forums or submissions of written feedback (Adams, 2022). **Product makers:** Can provide input through an open forum to allow for sharing perspectives, presenting relevant evidence, or raising concerns about the impact or implementation of the guideline (Eccles, 2012). **Providers:** Can provide input through an open forum to allow for sharing perspectives, presenting relevant evidence, or raising concerns about the impact or implementation of the guideline (Eccles, 2012). Consultations can occur through face‐to‐face meetings or online (Adams, 2022). Opinions can be sought through surveys, interviews, focus groups, online forums or submissions of written feedback (Adams, 2022). Include providers to increase the relevance of guidelines for allied health professionals to support the long‐term engagement of professional interest‐holders (Rapu, 2005). **Public:** The public should be engaged throughout the whole process (GIN, 2021). Collect public feedback about the scope of the guideline as well as after the evidence is summarized and the first and near final drafts are prepared (Kunz, 2012). Feedback can also be collected after the guideline is published (Kunz, 2012). At the outset, decide which stages will utilize public comment and create materials that facilitate meaningful engagement (GIN, 2021). The process for engagement should be tailored to suit the age, cognitive ability, and culture of the patients and adjustments should be made for patients with physical or sensory impairments (GIN, 2021). When engaging with underrepresented groups, such as children or people with severe mental illness, plan and tailor specific practical and informal support strategies and consider legislation, cognitive capacity, and illness fluctuations (GIN, 2021).
7. Conflict of Interest (COI) Considerations	**Patients:** Can review or assess the conflicts of interest of panel members (Armstrong, 2017). Patients should be asked about which conflicts of interest and mitigation strategies are most salient for a particular guideline (Grant, 2021).
8. (PICO) Question Generation	**Patients:** Can assess the ‘real world’ applicability of the questions as well as their relevancy and usefulness (Armstrong, 2017). Include patients on the systematic review team (GIN, 2021). Patients can help develop the questions, analytic framework, and research plan for the evidence review to ensure its scope has ‘real world’ applicability (Grant, 2021). Opinions can be sought through focus groups (Armstrong, 2017). **Public:** Solicit public comment on the questions (Armstrong, 2017). Include the public on the systematic review team (GIN, 2021).
9. Considering Importance of Outcomes and Interventions, Values, Preferences and Utilities	**Patients:** Can identify and define outcomes of relevance, can be asked to rate the importance of outcomes, suggest proxies and discuss their acceptability, suggest confounding factors, barriers and facilitators for specific aspects of care, and particular populations of interest or multimorbidities (Armstrong, 2017; Khodyakov, 2020; MacLennan, 2017). Patients can help develop the analytic framework (Grant, 2021). **Public:** Can identify outcomes of relevance, can be asked to rate the importance of outcomes, suggest proxies and discuss their acceptability, suggest confounding factors, and particular populations of interest or multimorbidities (Armstrong, 2017). The draft plan can be posted for public review (Armstrong, 2017).
10. Deciding what Evidence to Include and Searching for Evidence	**Patients:** Can suggest literature that describes patient preferences, additional search terms, confounding factors, and particular populations of interest (Armstrong, 2017). Conduct wide consultation to obtain feedback about the evidence used to inform the guidelines/recommendations (Fretheim, 2006). **Policymakers:** Conduct wide consultation to obtain feedback about the evidence used to inform the guidelines/recommendations (Fretheim, 2006). **Programme Managers:** Conduct wide consultation to obtain feedback about the evidence used to inform the guidelines/recommendations (Fretheim, 2006). **Providers:** Conduct wide consultation to obtain feedback about the evidence used to inform the guidelines/recommendations (Fretheim, 2006).
11. Summarizing Evidence and Considering Additional Information	**Patients:** Can review the evidence, can suggest alternate interpretations of the evidence and assess the believability of the results (Armstrong, 2017; MacLennan, 2017). **Payers of health services:** Can review the evidence (MacLennan, 2017). **Providers:** Can review the evidence (MacLennan, 2017). **Public:** Collect public feedback about the scope of the guideline and when the evidence has been summarized and for the first and final drafts (Kunz, 2012). Request feedback after the publication of the guideline (Kunz, 2012).
12. Judging Quality, Strength or Certainty of a Body of Evidence	**Patients:** Can assist with the critical appraisal of studies and the synthesis (Armstrong, 2017). Patients can appraise the degree to which the summaries and conclusions seem valid, meaningful, and intelligible (Grant, 2021). **Public:** Post the draft evidence summary for public comment (Armstrong, 2017).
13. Developing Recommendations and Determining their Strength	**Patients:** Can help with translating conclusions into clear and respectful recommendations, provide input when there are gaps in the evidence, indicate which recommendations are counter‐intuitive so that additional explanation can be provided (Armstrong, 2017). Consultation with patients helps ensure that their values have been integrated into the recommendations (Kelson, 2012). Patients can help develop recommendations that foster shared decision‐making, respect variation in patient perspectives, and identify gaps from a patient perspective (Grant, 2021). Patients can provide feedback on whether the recommendations are consistent with the range of their values and preferences and if they are practical for the ‘real world’ (Kelson, 2012). They can also rate the recommendations based on importance and acceptability (Khodyakov, 2020). **Public:** Draft recommendations can be posted for public comment (Armstrong, 2017).
14. Wording of Recommendations and of Considerations of Implementation, Feasibility and Equity	**Patients:** Can help with translating conclusions into clear and respectful recommendations, provide input when there are gaps in the evidence, indicate which recommendations are counter‐intuitive so that additional explanation can be provided (Armstrong, 2017). Consultation with patients helps ensure that their values have been integrated into the recommendations (Kelson, 2012). Patients can help develop recommendations that foster shared decision‐making, respect variation in patient perspectives, and identify gaps from a patient perspective (Grant, 2021). Patients can provide feedback on whether the recommendations are consistent with the range of their values and preferences and if they are practical for the ‘real world’ (Kelson, 2012). They can also rate the recommendations based on importance and acceptability (Khodyakov, 2020). **Public:** Draft recommendations can be posted for public comment (Armstrong, 2017).
15. Reporting and Peer Review	**Patients:** Conduct wide consultation to encourage feedback on the draft guidelines and recommendations (Fretheim, 2006; GIN, 2021; Wedzicha, 2011). Patients should also approve the patient version of the guideline (Wedzicha, 2011). **Policymakers:** Conduct wide consultation to encourage feedback on the draft guidelines and recommendations (Fretheim, 2006). **Product Makers:** Can invite members of industry to comment on drafts of the review (Kunz, 2012). **Programme Managers:** Conduct wide consultation to encourage feedback on the draft guidelines and recommendations (Fretheim, 2006). **Providers:** Conduct wide consultation to encourage feedback on the draft guidelines and recommendations (Fretheim, 2006). **Public:** Collect public feedback on the first and penultimate drafts of the guideline (GIN, 2021; Kunz, 2012).
16. Dissemination and Implementation	**Patients:** Consult with patients regarding dissemination and barriers/facilitators to implementation (Armstrong, 2017; Duff, 1996; Fretheim, 2006; GIN, 2021; Grant, 2021). Consider using social media to publicly thank the patient and public members who participated to allow them to showcase their involvement (GIN, 2021). Allow patients to help with media releases and promote awareness (GIN, 2021). Allow patients to engage with other patients to facilitate dissemination (Armstrong, 2017). Patients can endorse guidelines to improve their legitimacy and trustworthiness to their patient communities, nationally or internationally (Grant, 2021; MacLennan, 2017). **Policymakers:** Conduct wide consultation to encourage feedback on the dissemination plans and for supporting the adaptation and implementation of the guidelines and recommendations (Fretheim, 2006). **Programme Managers:** Conduct wide consultation to encourage feedback on the dissemination plans and for supporting the adaptation and implementation of the guidelines and recommendations (Fretheim, 2006). **Providers:** Conduct wide consultation to encourage feedback on the dissemination plans and for supporting the adaptation and implementation of the guidelines and recommendations (Fretheim, 2006). **Public:** Consider using social media to publicly thank the patient and public members who participated to allow them to showcase their involvement (GIN, 2021). Allow members of the public who have participated to help with media releases and promote awareness (GIN, 2021).
17. Evaluation and Use	**Patients:** Patients can link to their national and international communities to get opinions on priority‐setting and outcome measures to help contribute to the prioritization of future research (MacLennan, 2017). Collect feedback through questionnaires, focus groups from patients using the patient‐directed versions (GIN, 2021). **Public:** Collect public feedback regarding guidelines and set up a discussion board after its publication (Kunz, 2012).
18. Updating	**Patients:** Patients can help with updating and evaluating guidelines by assessing when guidelines need to be updated (Armstrong, 2017; Grant, 2021). Patients can define and prioritize changes in patient views of outcomes and interventions that may require a guideline update (Grant, 2021).

## 6 DISCUSSION

### 6.1 Summary of main results

This scoping review identified available guidance for interest‐holder engagement in guideline development. However, most of the guidance is focused on patient involvement. There is limited guidance for other interest‐holders, such as healthcare providers, policymakers, product makers, programme managers, and payers of health services. We did not identify any specific guidance for two of our groups: payers of health research and editors of peer‐reviewed journals. Surprisingly, there was limited guidance for healthcare providers and members of the public, even though involvement of these two groups is common in guideline development.

The available guidance focuses mainly on the early stages of guideline development, for example, identifying the scope of the guideline, and we found very little guidance for engaging with any of our identified interest‐holder groups related to judging the quality or certainty of the evidence, developing recommendations and considering implementation, feasibility and equity. Additionally, the studies were conducted in high‐income country settings and therefore may not reflect engagement within other contexts.

Many of our included papers included suggestions for the guideline commissioners or secretariat. For example, papers noted the importance of an experienced guideline panel chair who can ensure that each member of the panel has equal opportunities to contribute (GIN, 2021; Kunz, 2012). The appointed chair should have good facilitation skills and experience with effective conflict resolution. These issues are better described in the second review in this series which focuses on barriers and facilitators to interest‐holder engagement (Magwood et al., 2022).

This scoping review does not address the optimal timing of involvement of different interest‐holder groups and does not assess whether the recommendations from our included studies are appropriate or adequate. Specific guidance, related to the optimal number of guideline panel members, has not been provided as these decisions are dependent on the context and setting of the guideline as well as available time and resources. One paper reported that challenges may arise when a guideline panel has more than 15 members while another mentioned that larger groups can operate effectively (Eccles, 2012; Kunz, 2012). While larger groups may be more difficult to manage and require a skilled and experienced facilitator, they also offer more opportunity for diversity in opinions among members and therefore may be more reliable, may enhance credibility and lead to widespread acceptance and implementation of decisions and recommendations (Oliver et al., 2018). Regardless of the size of the panel, all contributions from all members should be valued equally (Rapu, 2005).

The review is limited by the focus on empirical research and not on handbooks produced by guideline developing organizations. A separate review identifies and summarizes the available guidance produced by organizations such as the World Health Organization (Khabsa et al. in development).

## 7 AUTHORS' CONCLUSIONS

This scoping review has identified gaps in the literature regarding guidance for engaging with interest‐holders in the different stages of the guideline and recommendation process. In particular, there are some groups, such as payers of health research and editors of peer‐reviewed journals, for which no guidance was identified by this review.

The available guidance identified in this scoping review will be used along with the findings of the review of barriers and facilitators (Magwood et al., 2022) and managing conflicts of interest (Khabsa et al., 2022) to inform the items included in the GIN‐McMaster Guideline Development Checklist Extension for Engagement (Petkovic et al., 2020). This checklist extension will include guidance for engaging with all 10 identified interest‐holder groups throughout all stages of guideline development.

## CONTRIBUTIONS OF AUTHORS

Conceiving the review: PT, VW, TWC, JP

Designing the review: JP, AR, VW, PT, TWC

Coordinating the review: JP, AR, PA

Writing the protocol: JP, PA, AR, JK, LL

Providing general advice on the review: All authors

Securing funding for the review: PT, VW, JP

Approved final version of the review: All authors.

## DECLARATIONS OF INTEREST

SG's spouse is a salaried employee of Eli Lilly and Company and owns stock. SG has accompanied his spouse on company‐sponsored travel. SVK acknowledges research funding from the UK Medical Research Council, Scottish Government Chief Scientist Office and an NRS Senior Clinical Fellowship. SVK is an honorary Consultant in Public Health at Public Health Scotland and has provided unpaid advice to Obesity Action Scotland, Scottish Government and UK Government. VW is editor‐in‐chief of the Campbell Collaboration. This review will be handled by an independent editor, and the co‐chairs of the relevant group will act in lieu of editor‐in‐chief. TWC has developed and published several peer‐reviewed publications that could potentially be included in the review. TWC currently holds one research contract with the Patient‐Centred Outcomes Research Institute and another with the Pharmaceutical Research and Manufacturers of America Foundation that address a similar topic. R.A.M. serves as a Board member for the Evidence Foundation, is a member of the medical advisory board for the National Kidney Foundation Chapter serving Western MO, OK, and KS, and is a counsellor for the Women In Nephrology (WIN). She receives research funding from the NIH, the WHO, the American College of Rheumatology, the American Society of Hematology. She serves as Chair of the Midwest Comparative Effectiveness Public Advisory Council of the Institute for Clinical and Economic Review (ICER). She was the site PI for the EMPA‐kidney study which is subaward from the Duke Clinical Research Institute. The study received grant funds from Boehringer Ingelheim. The grant funds went to the University of Kansas Medical Centre Research Institute and did not cover any of Dr. Mustafa's salary or compensation. This work is unrelated to this article.

## SOURCES OF SUPPORT

### Internal sources

- No sources of support provided.

### External sources

- Canadian Institutes of Health Research, Canada CIHR Project Grant.

## REGISTRATION AND PROTOCOL

Protocol was published with Campbell Systematic Reviews (Petkovic et al., 2022).

## Supporting information

Supporting information.

Supporting information.

Supporting information.

Supporting information.

Supporting information.

Supporting information.
