# Supplementary material for: Guidance for engagement in health guideline development: A scoping review
Source: Campbell Syst Rev. 2024 Nov 25;20(4):e70006. doi: 10.1002/cl2.70006 (PMC11586780; doi:10.1002/cl2.70006)
Supplement: Supplementary file 2 — Supporting information. [file CL2-20-e70006-s002.docx]

**Supplementary material 2: Characteristics of included studies**

**Studies ordered by Study ID**

| **Adams 2022** | | |
| --- | --- | --- |
| ***Study characteristics*** | | |
| Country | Australia, Denmark | |
| Stakeholder groups in author team | Principal investigators | |
| Stakeholder groups for which guidance is provided | Patients, Principal investigators, Providers, | |
| Study Design | Methodology paper | |
| Definition of stakeholder | "Within guideline development, stakeholders include those who have a legitimate interest in a guideline and/or who may affect, or be affected by it. Stakeholders may include health professionals, patients and their representatives, those financing, managing or monitoring care, employers, and manufacturers." | |
| Frequency/Mode/Level of engagement | Not reported | |
|  | | |
|  |  |  |
| **Armstrong 2017** | | |
| ***Study characteristics*** | | |
| Country | USA | |
| Stakeholder groups in author team | Principal investigators | |
| Stakeholder groups for which guidance is provided | Patients, Public | |
| Study Design | Methodology Paper | |
| Definition of stakeholder | "We use the term 'patient' to refer to all of the lay stakeholders filling this role, consistent with other frameworks" | |
| Frequency/Mode/Level of engagement | Not reported | |

| **Bjorkqvist 2021** | |
| --- | --- |
| ***Study characteristics*** | |
| Country | United Kingdom, the Netherlands |
| Stakeholder groups in author team | Principal investigators, payers/purchasers of health services |
| Stakeholder groups for which guidance is provided | Patients |
| Study Design | Methodology Paper |
| Definition of stakeholder | Not reported |
| Frequency/Mode/Level of engagement | Not applicable |
| **Chalmers 2017** | |
| ***Study characteristics*** | |
| Country | International |
| Stakeholder groups in author team | Patients, Providers |
| Stakeholder groups for which guidance is provided | Patients |
| Study Design | Case report |
| Definition of stakeholder | Not reported |
| Frequency/Mode/Level of engagement | Face-to-face engagement, decision-making role |

| **Duff 1996** | |
| --- | --- |
| ***Study characteristics*** | |
| Country | United Kingdom |
| Stakeholder groups in author team | Principal investigators |
| Stakeholder groups for which guidance is provided | Patients, |
| Study Design | Case report |
| Definition of stakeholder | Not reported |
| Frequency/Mode/Level of engagement | One-time, face-to-face seminar participation. Decision-making role. |
| **Eccles 2012** | |
| ***Study characteristics*** | |
| Country | International (Canada, UK, USA) |
| Stakeholder groups in author team | Principal investigators |
| Stakeholder groups for which guidance is provided | Patients, Payers/purchasers of health services, Providers, Product makers |
| Study Design | Methodology paper |
| Definition of stakeholder | Not reported |
| Frequency/Mode/Level of engagement | Not applicable |

| **English 2017** | |
| --- | --- |
| ***Study characteristics*** | |
| Country | Kenya, UK |
| Stakeholder groups in author team | Principal investigators, Policymakers |
| Stakeholder groups for which guidance is provided | Patients, Policymakers, Providers, Principal investigators |
| Study Design | Case report |
| Definition of stakeholder | "The second was to engage government personnel and those with a major potential role in promoting ownership and adoption of policy in a shared decision-making process" |
| Frequency/Mode/Level of engagement | Face-to-face, decision-making |
| **Fretheim 2006** | |
| ***Study characteristics*** | |
| Country | International (Italy, Norway) |
| Stakeholder groups in author team | Principal investigators |
| Stakeholder groups for which guidance is provided | Patients, Policymakers, Providers, Program managers |
| Study Design | Narrative review |
| Definition of stakeholder | "Stakeholders such as consumers, health professionals that work within the relevant area, and managers or policy makers." |
| Frequency/Mode/Level of engagement | Not applicable |

| **GIN 2021** | |
| --- | --- |
| ***Study characteristics*** | |
| Country | International |
| Stakeholder groups in author team | Patients, Principal investigators |
| Stakeholder groups for which guidance is provided | Patients, Providers, Public |
| Study Design | Methodology paper |
| Definition of stakeholder | "Patients and the public can refer to people with personal experience of a disease, condition or service (patients, consumers, users), their carers or family members, and people representing a collective group of patients or carers (representatives or advocates). It may also refer to members of society interested in health and social care services, or whose life is affected directly or indirectly by a guideline (citizens, taxpayers, the public)." |
| Frequency/Mode/Level of engagement | Not applicable |
| **Grant 2021** | |
| ***Study characteristics*** | |
| Country | USA |
| Stakeholder groups in author team | Principal investigators |
| Stakeholder groups for which guidance is provided | Patients |
| Study Design | Methodology paper |
| Definition of stakeholder | Not reported |
| Frequency/Mode/Level of engagement | Not reported |

| **Kelson 2012** | |
| --- | --- |
| ***Study characteristics*** | |
| Country | International |
| Stakeholder groups in author team | Principal investigators |
| Stakeholder groups for which guidance is provided | Patients, Providers |
| Study Design | Narrative review |
| Definition of stakeholder | "People with a legitimate interest in guideline development include those who deliver care (healthcare professionals), those who finance, commission, manage, and assure care (government, policymakers, healthcare providers, and regulators), those who develop and manufacture healthcare products, and those who receive care (consumers of healthcare)" |
| Frequency/Mode/Level of engagement | Not applicable |
| **Khodyakov 2020** | |
| ***Study characteristics*** | |
| Country | USA |
| Stakeholder groups in author team | Patients, Principal investigators |
| Stakeholder groups for which guidance is provided | Patients |
| Study Design | Methodology Paper |
| Definition of stakeholder | Not reported |
| Frequency/Mode/Level of engagement | Not repored |

| **Kunz 2012** | |
| --- | --- |
| ***Study characteristics*** | |
| Country | International |
| Stakeholder groups in author team | Principal investigators |
| Stakeholder groups for which guidance is provided | Patients, Program managers, Principal investigators, Product makers, Providers, Public |
| Study Design | Narrative review |
| Definition of stakeholder | Not reported |
| Frequency/Mode/Level of engagement | Not reported |
| **MacLennan 2017** | |
| ***Study characteristics*** | |
| Country | International |
| Stakeholder groups in author team | Principal investigators |
| Stakeholder groups for which guidance is provided | Patients, Payers/purchasers of health services, Providers |
| Study Design | Methodology Paper |
| Definition of stakeholder | "key stakeholders (eg, patients, carers, charitable organisations, health care funders)" |
| Frequency/Mode/Level of engagement | Not reported |

| **Rapu 2005** | |
| --- | --- |
| ***Study characteristics*** | |
| Country | UK |
| Stakeholder groups in author team | Providers |
| Stakeholder groups for which guidance is provided | Providers |
| Study Design | Case Report |
| Definition of stakeholder | "Professional stakeholders are national organisations (eg: RCN, COT, RCM) which represent frontline staff who provide the care described in the clinical guideline" |
| Frequency/Mode/Level of engagement | questionnaires (mail & email), interviews (face-to-face & telephone), advice/feedback role |
| **Wedzicha 2011** | |
| ***Study characteristics*** | |
| Country | UK |
| Stakeholder groups in author team | Principal investigators |
| Stakeholder groups for which guidance is provided | Patients, Public |
| Study Design | Methodology Paper |
| Definition of stakeholder | Not reported |
| Frequency/Mode/Level of engagement | Not reported |

**References to studies**

**Adams 2022 {published data only}**

- *Adams AN, Chamberlain D, Thorup CB, Grønkjær M, Conroy T. Ethical and feasible stakeholder engagement in guideline development. Collegian 2022;30(1):101-109.

**Armstrong 2017 {published data only}**

- Armstrong MJ, Mullins CD, Gronseth GS, Gagliardi AR . Framework for enhancing clinical practice guidelines through continuous patient engagement. Health Expectations 2017;20:3-10.

**Bjorkqvist 2021 {published data only}**

- *Björkqvist J, Giles RH, Cornford P, Makaroff LE, Van Hemelrijck M, Darraugh J, Cowl J, MacLennan S, MacLennan SJ. Providing a Framework for Meaningful Patient Involvement in Clinical Practice Guideline Development and Implementation. European Urology Focus 2021;7(5):947-950.

**Chalmers 2017 {published data only}**

- *Chalmers JD, Timothy A, Polverino E, Almagro M, Ruddy T, Powell P, Boyd J. Patient participation in ERS guidelines and research projects: the EMBARC experience. Breathe 2017;13(3):194-207.

**Duff 1996 {published data only}**

- *Duff LA, Kelson M, Marriott S, Mcintosh A, Brown S, Cape J, Marcus N, Traynor M. Clinical guidelines: involving patients and users of services. Journal of Clinical Effectiveness 1996;1(3):104-112.

**Eccles 2012 {published data only}**

- *Eccles MP, Grimshaw JM, Shekelle P, Schünemann HJ, Woolf S. Developing clinical practice guidelines: target audiences, identifying topics for guidelines, guideline group composition and functioning and conflicts of interest. Implementation Science 2012;7:60.

**English 2017 {published data only}**

- *English M, Irimu G, Nyamai R, Were F, Garner P, Opiyo N. Developing guidelines in low-income and middle- income countries: lessons from Kenya. Arch Dis Child 2017;102:846-851.

**Fretheim 2006 {published data only}**

- *Fretheim A, Schünemann HJ, Oxman AD. Improving the use of research evidence in guideline development: 3. Group composition and consultation process. Health Research Policy and Systems 2006;4:15.

**GIN 2021 {published data only}**

- *GIN. *GIN Public Toolkit*. Guidelines International Network 2021; Available from: https://g-i-n.net/wp-content/uploads/2023/07/Toolkit-combined.pdf.

**Grant 2021 {published data only}**

- Grant S, Armstrong C, Khodyakov D. Online Modified-Delphi: a Potential Method for Continuous Patient Engagement Across Stages of Clinical Practice Guideline Development. Journal of General Internal Medicine 2021;36:1746-1750.

**Kelson 2012 {published data only}**

- *Kelson M, Akl EA, Bastian H, Cluzeau F, Curtis JR, Guyatt G, Montori VM, Oliver S, Schünemann HJ. Integrating Values and Consumer Involvement in Guidelines with the Patient at the Center. Proceedings of the American Thoracic Society 2012 ;9(5):262-8.

**Khodyakov 2020 {published data only}**

- Khodyakov D, Grant S, Denger B, Kinnett K, Martin A, Peay H, Coulter I. Practical Considerations in Using Online Modified-Delphi Approaches to Engage Patients and Other Stakeholders in Clinical Practice Guideline Development. The Patient - Patient-Centered Outcomes Research 2020;13:11-21.

**Kunz 2012 {published data only}**

- *Kunz R, Fretheim A, Cluzeau F, Wilt TJ, Qaseem A, Lelgemann M, Kelson M, Guyatt G, Schünemann HJ. Guideline Group Composition and Group ProcessesArticle 3 in Integrating and Coordinating Efforts in COPD Guideline Development. An Official ATS/ERS Workshop Report. Proceedings of the American Thoracic Society 2012;9(5):229-33.

**MacLennan 2017 {published data only}**

- *MacLennan SJ, MacLennan S, Bex A, Catto JWF, De Santis M, Glaser AW, Ljungberg B, N'Dow J, Plass K, Trapero-Bertran M, Van Poppel H, Wright P, Giles RH. Changing Current Practice in Urology: Improving Guideline Development and Implementation Through Stakeholder Engagement. European Association of Urology 2017;72(2):161-163.

**Rapu 2005 {published data only}**

- *Rapu C. Enhancing Involvement in NICE guideline development. Nursing Standard 2005;20(2):48-50.

**Wedzicha 2011 {published data only}**

- *Wedzicha W, Fletcher M, Powell P. Making ERS guidelines relevant and accessible: involving patients and the public. Breathe 2011;8:9-11.
