# Supplementary material for: Guidance for engagement in health guideline development: A scoping review
Source: Campbell Syst Rev. 2024 Nov 25;20(4):e70006. doi: 10.1002/cl2.70006 (PMC11586780; doi:10.1002/cl2.70006)
Supplement: Supplementary file 3 — Supporting information. [file CL2-20-e70006-s006.docx]

| **Study** | **Reason for exclusion** |
| --- | --- |
| Brouwers 2018 | Not guidance, study of models of engagement |
| Devlin 2018 | Not guidance, example of development |
| Dunning 2012 | Not guidance, example of development |
| Morgano 2020 | Not guidance, example of development |
| Schunemann 2019 | Not guidance, example of development |
| Shin 2014 | Not guidance - example of engagement |
| van der Ham 2016 | Not guidance, example of development |
| vanderWeijden 2018 | Not guidance, quality criteria for patient-decision aids based on guidelines |
| Weiss 2020 | not guidance, example of development |
| Wiercioch 2020 | Not guidance, example of development |
| Wise 1995 | Guideline adaptation |

**References to studies**

**Brouwers 2018 {published data only}**

- Brouwers MC, Vukmirovic M, Spithoff K, Zwaal C, McNair S, Peek N. Engaging Cancer Patients in Clinical Practice Guideline Development: A Pilot Study. Curr. Oncol. 2018;25(4):250-256.

**Devlin 2018 {published data only}**

- Devlin JW, Skrobik Y, Rochwerg B, Nunnally ME, Needham DM, Gelinas C, Pandharipande PP, Slooter AJ, Watson PL, Weinhouse GL, Kho ME, Centofanti J, Price C, Harmon L, Misak  C, Flood P, Alhazzani W. Methodologic Innovation in Creating Clinical Practice Guidelines: Insights From the 2018 Society of Critical Care Medicine Pain, Agitation/Sedation, Delirium, Immobility, and Sleep Disruption Guideline Effort. Critical Care Medicine 2018;46(9):1457-1463.

**Dunning 2012 {published data only}**

- Dunning T, Savage S, Duggan N, Martin P. Developing clinical guidelines for end-of-life care: blending evidence and consensus. Int J Palliat Nurs. 2012;18(8):397-405.

**Morgano 2020 {published data only}**

- Morgano GP, Fulceri F, Nardocci F, Barbui C, Ostuzzi G, Papola D, Fatta LM, Fauci AJ, Coclite D, Napoletano A, De Crescenzo F, D'Alò GL, Amato L, Cinquini M, Lannone P, Schünemann HJ, Scatonni ML. Introduction and methods of the evidence-based guidelines for the diagnosis and management of autism spectrum disorder by the Italian National Institute of Health. Health and Quality of Life Outcomes 2020;18(1):81.

**Schunemann 2019 {published data only}**

- Schünemann HJ, Lerda D, Dimitrova N, Alonso-Coello P, Gräwingholt A, Quinn C, Follmann M, Mansel R, Sardanelli F, Rossi PG, Lebeau A, Nyström L, Broeders M, Ioannidou-Mouzaka L, Duffy SW, Borisch B, Fitzpatrick P, Hofvind S, Castells X, Giordano L, Warman S, Saz-Parkinson Z. Methods for Development of the European Commission Initiative on Breast Cancer Guidelines. Annals of Internal Medicine 2019;171(4):273-280.

**Shin 2014 {published data only}**

- *Shin JJ. Involving Stakeholders in the Development of Clinical Practice Guidelines. Otolaryngology-head and neck surgery : official journal of American Academy of Otolaryngology-Head and Neck Surgery 2014;150(6):907-9.

**van der Ham 2016 {published data only}**

- van der Ham AJ, van Erp N, Broerse JEW. Monitoring and evaluation of patient involvement in clinical practice guideline development: lessons from the Multidisciplinary Guideline for Employment and Severe Mental Illness, the Netherlands. Health Expectations 2016;19(2):471-482.

**vanderWeijden 2018 {published data only}**

- *van der Weijden T, Dreesens D, Faber MJ, Bos N, Drenthen T, Maas I, Kersten S, Malanda U, van der Scheur S, Post H, Knops A. Developing quality criteria for patient-directed knowledge tools related to clinical practice guidelines. A development and consensus study. Health Expect 2018;00:1-8.

**Weiss 2020 {published data only}**

- Weiss SL, Peters MJ, Alhazzani W, Agus MSD, Flori HR, Inwald DP, Nadel S, Schlapbach LJ, Tasker RC, Argent AC, Brierley J, Carcillo J, Carrol ED, Carroll CL, Cheifetz IM, Choong K, Cies JJ, Cruz AT, De Luca D, Deep A, Faust SN, De Oliveira CF, Hall MW, Ishimine P, Javouhey E, Joosten KFM, Joshi P, Karam O, Kneyber MCJ, Lemson J, MacLaren G, Mehta NM, Møller MH, Newth CJL, Nguyen TC, Nishisaki A, Nunnally ME, Parker MM, Paul RM, Randolph AG, Ranjit S, Romer LH, Scott HF, Tume LN, Verger JT, williams EA, Wolf J, Wong HR, Zimmerman JJ, Kissoon N, Tissieres P. Surviving sepsis campaign international guidelines for the management of septic shock and sepsis-associated organ dysfunction in children. Intensive Care Med 2020;46(Suppl 1):S10-S67.

**Wiercioch 2020 {published data only}**

- Wiercioch W, Nieuwlaat R, Akl EA, Kunkle R, Alexander KE, Cuker A, Rajasekhar A, Alonso-Coello P, Anderson DR, Bates SM, Cushman M, Dahm P, Guyatt G, Iorio A, Lim W, Lyman GH, Middeldorp S, Monagle P, Mustafa RA, Neumann I, Ortel TL, Rochwerg B, Santesso N, Vesely SK, Witt DM, Schünemann HJ. Methodology for the American Society of Hematology VTE guidelines: current best practice, innovations, and experiences. Blood Adv 2020;4(10):2351-2365.

**Wise 1995 {published data only}**

- *Wise, CG; Billi JE. A model for practice guideline adaptation and implementation: Empowerment of the physician. Journal on QUality Improvement 1995;21(9):465-476.
