# Supplementary material for: Guidance for engagement in health guideline development: A scoping review
Source: Campbell Syst Rev. 2024 Nov 25;20(4):e70006. doi: 10.1002/cl2.70006 (PMC11586780; doi:10.1002/cl2.70006)
Supplement: Supplementary file 4 — Supporting information. [file CL2-20-e70006-s003.zip › CA000252-data-package-info.html]

Data package 

# Supplementary material 4 to: Guidance for stakeholder engagement in health guideline development: A scoping review

Petkovic J, Riddle A, Lytvyn L, Khabsa J, Akl EA, Welch V, Magwood O, Atwere P, Graham ID, Grant S, John D, Katikireddi SVittal, Langlois EV, Mustafa RA, Todhunter-Brown A, Schünemann H, Stein AT, Concannon TW, Tugwell P  
https://doi.org/10.1002/14651858.CA000252

The material in this section has been supplied by the author(s) for publication under a Licence for Publication and the author(s) are solely responsible for the material. Cochrane has reviewed this material, but Cochrane has not copyedited, formatted or proofread. Cochrane accordingly gives no representations or warranties of any kind in relation to, and accepts no liability for any reliance on or use of, such material.

Back to top

# Data package

This data package conforms to version 2.1 of the Cochrane review data package specification.

Depending on the nature of the review, this data package may contain one or more of the following elements:

- Analysis data:
  - Overall estimates and settings (analysis level)
  - Subgroup estimates (mid-level estimates for each subgroup)
  - Individual data rows (all the rows in all the analyses)
  - Parameters for analyses (unique to DTA reviews)
- Study data:
  - Study information (information on which studies exist and study characteristics)
  - Study arms (arms reported for the study in the review)
  - Study references
  - Study results data (outcome data reported for the study in the review)
  - Risk of bias (judgements and support for judgements)
  - Study test data (all test data rows, unique to DTA reviews)
- Other references:
  - Additional references
  - References to other versions of the review
  - References awaiting classification

For more information on how to interpret and use the contents of this data package, please refer to the user guide.
