# Supplementary material for: Guidance for engagement in health guideline development: A scoping review
Source: Campbell Syst Rev. 2024 Nov 25;20(4):e70006. doi: 10.1002/cl2.70006 (PMC11586780; doi:10.1002/cl2.70006)
Supplement: Supplementary file 5 — Supporting information. [file CL2-20-e70006-s001.docx]

**Supplementary file 5: Data extraction items**

Extractor initials

Ref ID

Search source

Author and Year

Title

Country

Funding source

Author group - which stakeholders

Conflicts of interest declared?

Type of publication

Guideline topic

Definition of stakeholders

Stakeholder groups included (list all Ps)

Stakeholder roles (before, during, after)

Methodsof engagement

Frequency of engagement

Level of engagement

PROGRESS+ described?

Training provided to stakeholders

Stage 1 - Organization, budget, planning, and training (extract verbatim text)

Stage 2 - Priority Setting (extract verbatim text)

Stage 3 - Guideline Group Membership (extract verbatim text)

Stage 4 - Establishing guideline group processes (extract verbatim text)

Stage 5 - Identifying target audience and topic selection (extract verbatim text)

Stage 6 - Consumer and stakeholder involvement (extract verbatim text)

Stage 7 - Conflict of interest considerations (extract verbatim text)

Stage 8 - Question formulation (extract verbatim text)

Step 9 - Considering importance of outcomes and interventions, values, preferences and utilities (extract verbatim text)

Step 10 - Deciding what evidence to include and searching for evidence (extract verbatim text)

Step 11 - Summarizing evidence and considering additional information (extract verbatim text)

Step 12 - Juding quality, strength or certainty of a body of evidence (extract verbatim text)

Step 13 - Developing recommendations and determining their strength (extract verbatim text)

Step 14 - Wording of recommendations and of considerations about implementation, feasibility, and equity (extract verbatim text)

Step 15 - Reporting and Peer Review (extract verbatim text)

Step 16 - Dissemination and Implementation (extract verbatim text)

Step 17 - Evaluation and use (extract verbatim text)

Step 18 - Updating (extract verbatim text)

Are the key items/guidance clearly specified?

Can the guidance be applied easily without the need to search for additional information?

Can the guidance be adequately operationalised?

Does the guidance thoroughly describe information related to adaptation in a new setting?/Does the guidance thoroughly describe information related to evaluation in a new setting?

Can the guidance be applied to different types of guidelines

Can the guidance be used in different settings?

Can the guidance be adapted to additional stakeholders?

Does the guidance describe its underlying theory and principles?

Does the guidance describe a rigorous development process (such as a comprehensive literature review and/or a consensus-based methodology)
